# Development and Unfolding of the Life Course Movement in the Field of Maternal and Child Health: An Oral History

**DOI:** 10.1007/s10995-024-03938-y

**Published:** 2024-07-16

**Authors:** Alessandra N. Bazzano, Cheri Pies, Michael C. Lu, Padmini Parthasarathy, Amy Fine, Milton Kotelchuck

**Affiliations:** 1 Department of Social, Behavioral, and Population Sciences; Center of Excellence in Maternal Child Health, Tulane School of Public Health and Tropical Medicine, New Orleans, USA; 2 University of California Berkeley, Berkeley, USA; 3 Samya Strategies, Martinez, CA USA; 4 Health Policy Program Consultant, San Francisco, CA USA; 5 Harvard Medical School, Department of Pediatrics, Division of General Academic Pediatrics Mass General Hospital for Children, Boston, USA

**Keywords:** Maternal health, Child health, Life course, History, Social determinants of health

## Abstract

**Introduction:**

A life course perspective in maternal, child, and family health allows for integrated exploration of outcomes, incorporating multifactorial determinants of health to interrogate sources of inequity and identify opportunities for intervention. This article explores the historical development, integration, and implications of the contemporary life course perspective in the field of maternal and child health (MCH), and particularly the people and events which institutionalized the framework as central to national and local MCH practice and research over the last decades.

**Methods:**

Drawing on an oral history approach, key leaders of the life course movement in MCH were interviewed. Lived experiences and personal recollections of six interviewees were recorded and synthesized using a narrative descriptive approach to portray the social ecology of the movement’s origins.

**Results:**

We documented systematic efforts made in the first two decades of the 21st century to consciously promote life course through convening a National MCH Life Course Invitational Meeting, incorporating life course as a foundational framework for strategic planning at the Maternal Child Health Bureau, and development of tools and resources by MCH professional organizations.

**Discussion:**

The integration of life course theory into the MCH field signified a major shift towards addressing protective and social factors, which aligns with the field’s historical emphasis on social justice and rights-based approaches, and parallels the broader public health movement towards social determinants of health and the need to address structural racism. The ongoing relevance of the life course approach in promoting reproductive justice and addressing inequities in health underscores the historical importance of its adoption and use in the current mainstream of MCH research, policy, and practice.

## Significance

This historical overview details the development and expansion of the life course perspective in maternal and child health over the last two decades. It underscores the contributions of life course champions across MCH practice, policy, and research domains. Early advocates of a life course framework promoted responsive and adaptive strategies addressing the root causes of health inequity. This movement has led to a more comprehensive approach for reproductive, maternal, child, adolescent, and family health.

## Introduction

The life course perspective recognizes that health outcomes are shaped by a complex interplay of factors (biological, social, economic, environmental, behavioral) that contribute to health status both within an individual’s lifetime and across generations. By focusing on risk and protective factors impacting health development longitudinally, and how these relate to longer term health outcomes, the life course perspective provides a lens for understanding root causes of health disparities and for developing more effective interventions in MCH specifically and public health more broadly.

Much progress has been made to integrate the life course paradigm in the field of MCH in the United States, including the core concept that health comprises a multifaceted trajectory impacted by social determinants, risk, and protective factors that work across generations. The first author (Bazzano) is a maternal child health researcher and advocate leading federally funded training programs for graduate students and early career researchers who will comprise the MCH workforce of the future. Because the life course approach is central to MCH research and practice, the first author developed a graduate-level course focused on the theory for MPH students as a core requirement.

Given that the visionary nature, spread, and acceptance of the life course approach over the last two decades has been a key transformation in the MCH field, an article about its historical origins and impact seemed important as a contribution to the *Maternal and Child Health Journal’s* commemoration of the 100th anniversary of the MCH Section. Following exchanges between the first author and two advocates for the life course perspective—authors Kotelchuck and Pies—it was determined that the best way to document the history and impact of life course theory would be through oral history, by interviewing key leaders in the life course movement. This paper presents history, experiences, and recollections of several individuals instrumental in the development and elaboration of life course in MCH during the first two decades of the 21st century.

## Methods/Aims

An oral history approach was chosen as the method for gathering information on the introduction and spread of a life course orientation in the MCH field. Oral history is a method for creating and preserving first-hand, historically interesting information directly from people who experienced it (Oral History Association, 2018). The unique contribution of oral history lies in the potential to contextualize individual experiences within a wider social and historical framework. Interviews were conducted by the first author, an experienced qualitative researcher.

Participants were selected based on two of the senior authors’ (Kotelchuck and Pies) recommendations, and through a review of literature related to life course in MCH research, policy and practice from 2000 to 2020. Those interviewed (with the date of the interview) included: Cheri Pies DrPH, MSW (August 31, 2022), Milt Kotelchuck PhD, MPH (December 16, 2022), Amy Fine MPH (September 27, 2022), Michael Lu MD, MS, MPH (November 21, 2022), Padmini Parthasarathy MPH (October 4, 2022), and Neal Halfon MD, MPH (April 5, 2023).

Interviews were arranged by e-mail correspondence and conducted via Zoom teleconference; they were recorded and professionally transcribed. The interviews described here are archived and may be accessed upon request. The first author selected quotes from interviews to be included, and interviewees reviewed and approved them during manuscript development. The results section includes historical information noted by the interview participants, with additional references, as well as participants’ own recollections of key events and turning points in the evolution of contemporary life course approach in MCH.

## Results

### Emergence of Life Course into the MCH Field: Setting the Stage

Fields related to maternal and child health, particularly sociology, developmental psychology, chronic disease epidemiology, and health causation and disparities research, had long utilized life course models which influenced the development of this approach in MCH. With roots in sociology (Elder et al., 2003), where it was used for decades in research and practice, life course application in public health and MCH has centered on individual’s social context and cumulative health impacts. This complemented life span approaches such as those used by developmental psychologists (Bronfenbrenner & Ceci, 1994) and others focused on adaptation, plasticity, and how individual differences developed via interactions with their environments. These strands came together through linked lives and other studies that explored developmental pathways (Brooks-Gunn et al., 1993). Life course approaches focused on population health in chronic disease epidemiology were important in development of the current MCH life course approach (Ben-Shlomo & Kuh, 2002), as were British cohort studies (Barker, 1990; Marmot et al., 1997), biological embedding studies (Hertzman & Boyce, 2010), as well as research focused on Developmental Origins of Health and Disease, or DoHAD (Gluckman et al., 2008).

Interestingly, the work of numerous researchers, practitioners, and communities to develop and embrace life course theory in MCH can be seen to have reinvigorated historical dialogues about health equity, economic disparities, and social determinants which reflect the origins of MCH programs beginning with the Children’s Bureau (Bradbury & Eliot, 1956).

The first studies conducted by the Children’s Bureau at its creation in 1913 were devoted to understanding the causes of infant mortality. These studies focused not solely on biological causes and medical care, but also on factors such as a father’s employment earnings and other social and familial conditions existing before the birth of the child. One report stated that “community action can remedy many conditions dangerous to infants” while another Bureau study on maternal mortality included recommendations on surviving various social determinants of health (Bradbury & Eliot, 1956). These Children’s Bureau studies were used to advocate for improving hygiene and sanitation more broadly, to expand pasteurization of milk, to legislate for minimum wages and pensions, and to address risk and protective factors for life course health (Bradbury & Eliot, 1956).

However, despite these early studies on social determinants, and the collapse of the historic 1921 Sheppard–Towner Maternity and Infancy Protection Act, more clinical and medical approaches to improving infant and maternal mortality and morbidity dominated MCH research and practice in the US. For decades, care for women and children would primarily be provided through physicians and clinics, with limited preventive health services (Bradbury & Eliot, 1956; Lesser, 1985).

By the 1980’s the MCH field experienced another national focus on infant mortality reduction which emphasized both increased Medicaid access to prenatal care and comprehensive prenatal care with an enhanced focus on nutrition, health education, social work, and home visiting (Kotelchuck, 2018). In the intervening decade, many MCH public health agencies transitioned from delivery of clinical care to more population based, systems-focused prevention and health improvement strategies recognizing social context and determinants of health before and after pregnancy (Walker, 2003).

A similar awareness of social context was also emerging in this period in the broader public health field regarding inequities in reproductive outcomes between Black/African American populations and White populations. Diane Rowley, a physician-researcher, and author of another paper within this MCH history special issue (Rowley et al., 2023), led a team of epidemiologists at CDC from the early 1990s in studies on the experiences of Black MCH populations (Rowley, 1994). Her work highlighted contributions beyond biologic determinants and focused on non-medical, social factors such as racism, poverty, limited access to health care services, and other factors related to inequitable health outcomes (Rowley, 2001). Rowley stressed the importance of social, cultural, and political context for Black/African American women; environmental stressors and physiologic responses; and protective mechanisms in communities. These topics would be highlighted in subsequent years through life course approaches.

Despite scientific advancements in obstetrical and neonatal care and a persistent emphasis on access to prenatal care in maternal health efforts, the field of MCH had seen little progress on improving equity in the early 2000’s. Kotelchuck stated:

In 2002, we were basically a prenatal care medical model, and there were critiques coming up saying it’s not really the answer… there were people saying if you did one prenatal care visit before someone got pregnant, a preconception visit, that was the solution, but we were only still getting to people too late….

Since health outcomes were not improving (Guyer, 2001) many MCH programs made a shift to systems level social determinants of health approaches. Several Title V programs in the 1990s (e.g., Massachusetts and Washington) were using population-based policy and systems strategies, in addition to individual and/or clinical approaches to improving birth and other health outcomes. These included a social determinants of health and lifespan approach. States that did this had some of the best outcomes in the 1990s including lowering infant mortality rates in underserved communities and the guidance for the Title V block grant also supported these system level approaches, effectively set the stage for the nascent life course perspective to emerge in the field of MCH (Walker, 2003).

Pediatrician Neal Halfon’s work helped to catalyze this shift, and to sustain it over the next 30 years. His extensive work with children in the foster care system in the 1980s and a background in epidemiology and clinical scholarship provided insights into the growth and health trajectories of young children exposed to trauma. His intellectual journey over the next two decades of analyzing and synthesizing clinical and research experiences with the growing understanding of the importance longitudinal research, neurobiology of early childhood experiences, and developmental aspects of health, led to his initial seminal article on life course health development (Halfon & Hochstein, 2002). The Life Course Health Development Framework proposed by Halfon and Hochstein, were attempts to integrate prior streams of work on life course with newer findings from epigenetics and neurobiology. Halfon described the dominant approaches at the time, and the impetus for change:

The challenge was that all of the health-related quality of life research that was coming out at that time… all stated that everybody started off with perfect health and that it was a decay function. I [felt] that’s wrong. There’s a developmental process that leads to plateau and you actually have different plateaus. And you don’t need a health maintenance organization, you need a health development organization. So, the first paper that I wrote was in Milbank about the health development organization. Then I sat down, and I wrote the Life Course Health Development, to synthesize this into one model. I wanted to do this not just for research purposes, but so it could be applied to how we design our data systems, how we approach prevention.

Around the time that Halfon was developing the life course health development framework, Michael Lu, an academic-hospital based obstetrician and a colleague of Halfon at UCLA, recognized that the research at the time did not sufficiently explain severe MCH racial health disparities. Lu concluded, as Rowley and others (Blackmore et al., 1993) had noted, that factors beyond provision of medical care were crucially important, setting him on a path which would shape not only his career but the future of the MCH field. Lu recognized that previous attempts to improve birth outcomes and reduce disparities through clinical interventions, such as increased prenatal care, medical homes, and better deliveries–while necessary and valuable– were not sufficient to address glaring disparities in birth outcomes. As Lu stated:

What was most distressing to those who did research in this area was that [identified] risk factors explained less than 10% of variance in birth weight…that’s where we were really after decades of research. All we could explain was about 10%. So that’s when my friend, Neal Halfon at UCLA, introduced me to the life course perspective, and he had been using the life course perspective to look at child health outcomes. He said, ‘Why don’t we look at disparities in birth outcomes from a life course perspective?’

Lu made numerous presentations throughout the country both in academic and practice settings, articulating his evolving understanding of reproductive inequities through the lens of life course theory and advocating for changing MCH programming beyond clinical care at state and local levels. Lu’s contention, that you can’t cure a lifetime of ills in nine months of a pregnancy, became the central statement of the evolving MCH life course perspective. Within the pediatric community, there was much interest in developmental pediatrics and social determinants related to the cumulative effects of childhood poverty and trauma on children’s social, emotional, educational and health development. These viewpoints were summarized in the influential publication *Neurons to Neighborhoods* (National Research Council and Institute of Medicine, 2000). According to Kotelchuck, Lu in part conceptually utilized pediatric cumulative life course ideas to understand reproductive outcomes, and expanded ideas from the women’s health movement from a focus on the time just before pregnancy to encompass a lifetime of health experiences.

### Development of Life Course MCH Theory

The seminal article by Michael Lu and Neal Halfon (2003), *Racial and ethnic disparities in birth outcomes: a life course perspective*, formally introduced an evolving life course model to the MCH field. The article synthesized longitudinal models describing developmental origins of adult disease, as well as cumulative pathways, calling for research on racial disparities in birth outcomes ‘to examine differential exposures to risk and protective factors not only during pregnancy, but over the life course’ (Lu & Halfon, 2003). The aim of this new approach was to promote identification of interventions and policy to eliminate disparities; the article complemented Halfon’s earlier article (Halfon & Hochstein, 2002) which laid out a developmental framework to life course health, addressing policy and research. Lu studied and discussed the life course perspective over the next decade, not only in academic settings but also in practice settings, where the concepts were translated into MCH programming at state and local levels. For him, the drive to develop the life course perspective was partly rooted in frustration about the inability to ascertain the causes of persistent racial and ethnic disparities in birth outcomes. As Lu recalled:

When we started we really didn’t have any idea where this was going. We were just at a crossroads where we thought the old explanations didn’t explain very much. In that initial article we synthesized areas of emerging science at that time…developmental origins of adult health and disease…with a growing science of epigenetics and environmental influences…and cumulative pathways best known as weathering and the concept of allostatic load.

Since Kotelchuck was aware of how important the new framework was to the MCH field, he wrote a supportive editorial accompanying the seminal paper challenging the MCH field to adopt this new paradigm and change its practice models (Kotelchuck, 2003). Kotelchuck stated:

We today take [life course] for granted because I believe the paradigm has shifted in our field, but at that time you have to remember this was pretty radical… it was a very disruptive idea…[obstetricians] basically couldn’t see how they could actually do anything before the woman showed up in their doorway.

Yet, returning to an emphasis on prevention and addressing social determinants in the field of MCH through the life course perspective was a logical link back to MCH roots. As Kotelchuck noted:

The differential impact of social factors increasing or decreasing your health trajectory and its differential impact on birth outcomes—I believed, I felt it was correct—but the reason I liked it is that for me, it allowed a re-discussion of social factors back into the MCH community.

But even as the life course approach was becoming more widely accepted by many in the MCH field, others provided important cautionary critiques. For example, pediatrician Wise (2003) noted the potential for misinterpreting life course frameworks and casting early-life influences and interactions as deterministic rather than influential. He cautioned that early-life interactions had varying causes and timing which could affect whether they could be addressed through interventions later in life. Wise’s work warned that focusing on early exposures could diminish the impact of events in adolescence and adulthood or set up a zero-sum game for applying resources earlier in the lifespan versus later. Instead, Wise proposed a “human-capacity” model of the life course that linked the investigation of underlying fundamental mechanisms with an analysis of the relative efficacy of influences and policies at various developmental stages (Wise, 2009).

Later critiques included that life course as a *perspective* does not provide a clear framework for action, without ontological and epistemological grounding, nor clear definitions of terms, concepts, and variables that could lead to actionable research or transformational change in programs, policies, or practices (Halfon, personal communication). Notwithstanding concerns, adoption of the perspective gained ground.

### From Theory to Action

Cheri Pies, a public health practitioner and academic, recognized that public health professionals at state and local health departments could also benefit from the life course approach, since they witnessed, in their work with communities, key issues used in the new framework: economic and social issues, racism, and environmental concerns. Pies described a crucial ‘aha’ moment when she heard Michael Lu giving a talk at the time.

I heard him speak, and he spoke so beautifully with a very clear agenda and unfolding in his lecture about the influence of social determinants of health on birth outcomes, with an understanding that these determinants weren’t just for the individual woman at that moment in time, but in fact were cumulative. That came not only from her life course, but the life course of her predecessors, her mother, her grandmother, and the like. He contextualized it in such an accessible way that it hit me–I could be thinking about this not in a theoretical way, but as a practical approach. I went and heard him speak with the director of public health at the time at Contra Costa County, Wendell Brunner, who was himself an activist and a visionary. When I said to Wendell, ‘I want to try to do something that would encapsulate life course into our programs’ he said, ‘Give it a try. See what you can come up with.’

As the head of the Family, Maternal and Child Health (FMCH) Programs at the county public health agency, Contra Costa Health, in California, Pies launched in 2005 the first MCH Life Course Initiative to respond to high rates of low birth weight and infant mortality, especially among Black/African American populations, despite access to quality prenatal care. One program from the initiative was called Building Economic Security Today (BEST), which aimed to reduce inequities in maternal child health outcomes for low-income Contra Costa families by improving their financial security and stability (Parthasarathy et al., 2014). Guided by the life course approach, the program nonetheless required careful introduction in a field previously dominated by a focus on prenatal care. Pies recalled:

We felt that it was really the right direction to go. Particularly in our WIC program, women were excited about [financial literacy] and they wanted to talk more, and they wanted help setting up a bank account, a savings account, for example. I think that the tide was starting to change at that time when people were talking much more actively about social determinants of health. Some [staff] were right on board right away. Others were like, ‘This is just more work. Is this really going to make a difference?’ Not in a negative way, just ‘What? Something new?’

Padmini Parthasarathy, a public health practitioner, who led the BEST program with Pies recalled it this way:

The main lesson, along with needing buy-in, was that any kind of change takes a lot of time. Even with the earlier general life course perspective training and education, the reason we did it for three years [at Contra Costa Health] was, you need to hear things more than once…it was just such a different time. People were not talking about social determinants and race and poverty and health all the time. The science was there. It’s not like it was new, but it wasn’t the kind of overriding or overarching philosophy as it is now.

The training work that Pies and Parthasarathy did was later disseminated in an article in this journal (Pies et al., 2012). As momentum for the approach grew, Pies said:

…there was starting to be more conversation in the field of public health about racism, equity, equality that was generated. Once these theoretical concepts started to be discussed at gatherings, at meetings, I think people start to tell their own stories. I think those stories influenced the ways in which people understood the broader connections.

### Spreading the Life Course Message

In the late 2000s, as Kotelchuck recalled, the advocates of life course perspective were conscious of the dominance of, and investments in, more traditional models to improve infant mortality in the MCH field. They recognized that a conscious and strategic effort was needed to incorporate the life course perspective into the mainstream of MCH. This sustained effort involved multiple strategies and actions, several of which are highlighted below.

#### Early Champions Convene a National MCH Life Course Invitational Meeting

In 2008, five years following the publication of Lu and Halfon’s original article, a National Maternal and Child Health Life Course invitational meeting was organized and convened in Oakland, CA by Milt Kotelchuck Michael Lu, Padmini Parthasarathy, and Cheri Pies. This meeting, funded by The California Endowment and supported by Contra Costa Health, was the first national meeting on MCH life course strategies, which fostered the use of life course theory by strengthening leadership capacity to advocate for these approaches. The meeting brought together 25 community, state, and national MCH leaders who were early adopters of life course theory in order to catalyze the growing momentum (Pies et al., 2009). Parthasarathy recalled that the organizers worked to have a cross section of participants from practice, policy, and research from across the country.

Over two and a half days of intensive discussions, participants sought answers about how to apply the life course perspective to five domains of MCH (theory, research, practice, policy, and education/training), spending half a day on each. The participants identified multiple action steps, including mapping the current landscape of initiatives, utilizing health equity as a guiding principle, developing life course-related materials for training, and supporting changes in policy. The meeting attendees also emphasized the importance of educating policymakers responsible for funding Title V programs about the value of integrating the life course perspective and promoting a framework based on the life course approach at national, state, and local levels.

Following the 2008 meeting, a series of publications detailing policy and practice avenues for integration of life course approaches into the field of MCH were prepared (Fine et al., 2009). According to Kotelchuck, within a few years, MCH life course began to be seemingly everywhere.

#### MCHB Incorporates Life Course as a Foundational Framework for Strategic Planning

The life course perspective also gained increased attention through the leadership efforts of Peter Van Dyck, the former Associate Administrator of the Maternal and Child Health Bureau. He influenced the adoption of the perspective broadly throughout the MCH Bureau and the larger field through his advocacy for the position; it was also included as the basis for the Bureau’s strategic planning efforts starting in 2009 (Fine & Kotelchuck, 2010). During interviews, Fine recalled:

Peter’s leadership was essential. He invited Neal Halfon, Michael Lu, and other early architects of MCH life course to come to MCHB and educate the entire staff about this new perspective on health development and health disparities; he raised critical, clarifying questions (for example, how does life course perspective fit with social determinants of health, and theories of race equity?), and he provided the opportunity for each Division of MCHB to explore the best ways to incorporate it in its work going forward. In addition, he used MCHB’s 75th Anniversary celebration as an opportunity to “rethink MCH”, asking Milt and me to develop a concept paper that would kick off a new MCHB strategic planning process using life course as an organizing framework.

In developing the concept paper, Fine noted that she and Kotelchuck undertook meetings with every Division of the MCH Bureau separately; they worked with leadership and staff to strategize how the life course perspective could be strategically applied to their Division and the Bureau’s programs, implicitly encouraging them to adopt more MCH life course concepts in their programming and activities. Kotelchuck recalled that, Michael D. Kogan, Director of the Office of Epidemiology and Research at MCHB stressed the need to infuse the new life course perspective into his division’s own research and evaluation and throughout the larger MCH Epidemiology community nationally. Consequently, new life course measures and research instruments were developed and included in the National Survey of Children’s Health, as well as new life course material being included in the joint CDC MCHB Epidemiology training program, and in the MCH Epidemiology Committee of the MCH Section of the American Public Health Association (APHA) (Kogan et al., 2015). Ultimately HRSA/MCHB funded an initial MCH Life Course Measurement Network and subsequently MCH Life Course Intervention Network headed by Neal Halfon and described later in this article.

The 2010 concept paper by Fine and Kotelchuck, *Rethinking MCH: The Life Course Model as an Organizing Framework* (Fine & Kotelchuck), soon had an impact on the field. The paper provided a succinct overview of MCH life course theory and focused on how four key concepts–*timing, timeline, environment, and equity—*shaped MCHB strategic planning. Fine noted that the paper helped to focus life course strategies on place and place-based initiatives, providing examples from communities across the country. The paper also acknowledged and addressed some of the critiques of the theory, de-emphasizing determinism and asserting that reduction of risk factors and enhancement of protective factors to improve health and well-being was possible at any point, regardless of current or historical health status. Perhaps most significantly, per Kotelchuck, the concept paper provided a common unifying starting point for MCHB, and the broader community, to develop a life course agenda for change across the country.

While Van Dyck’s leadership efforts early on helped to encourage the adoption of a life course approach at the federal, state, and local levels, and set the foundation for future MCHB investments, recent articles describe additional life course investments made over the last two decades by the MCHB, indicating the integration of life course theory within the Bureau, and by extension, nationally into the mainstream of MCH policy and practice (Foney et al., 2022; Warren & Kavanagh, 2023).

#### MCH Professional Organizations Develop Life Course Tools and Resources

A third set of critical activities to spread the adoption of the life course theory was led by MCH professional organizations. CityMatCH (a national membership organization of city and county health departments’ maternal and child health programs and leaders representing urban communities in the United States), the Association of Maternal Child Health Programs (AMCHP), and the Association of Teachers of Maternal and Child Health (ATMCH) were instrumental in uptake of the new paradigm. Parthasarathy described her involvement in the creation of the MCH Life Course Toolbox in 2017, through a partnership between Contra Costa Health and CityMatCH, to provide resources for MCH organizations and practitioners to apply the theory to practice (CityMatCH, 2017). The organization also encouraged attendees at national and local meetings to engage in experiential life course learning through the Life Course Game, a game originally developed in a hand-drawn version by trainees of Cheri Pies. The game was later used with Contra Costa Health staff and subsequently professionally produced by CityMatCH, who added illustrative vignettes and lifelike scenarios to enhance learning (CityMatCH University of Nebraska Medical Center, 2012).

AMCHP developed a Life Course Indicators Project with the aim of establishing standardized metrics for state and local MCH programs. The indicators were developed as a framework for planning and evaluating projects using the life course approach; the framework encompasses a wide range of topics: health conditions, services, access and quality, family dynamics, community factors, economic well-being, and policies (each indicator associated with a specific data source). AMCHP’s Life Course Indicators Online Tool provided access to these indicators, accompanied by narrative and national estimates (AMCHP, 2014). Although there are no mandatory programs or policies that require the collection of data on the indicators, several states actively participated in the Life Course Indicators Technical Assistance Project through 2014. A collaborative decision-making process with state teams led to a “short list” comprising 13 indicators, which have been designated as high priority for implementation in MCH programming. Adjustments were made to the definitions of certain indicators and finalized in 2018.

An additional strategy used to disseminate the life course perspective has been through publications and presentations. During the early 2010’s, advocates spoke at conferences, state and local MCH meetings, and produced white papers to further expand the message and provide tools for action. In 2014, a Special Issue of the *Maternal and Child Health Journal*, entitled *Advancing MCH Life Course*, was released to continue efforts to build a learning community and share innovative and practical projects underway using life course perspectives (Pies & Kotelchuck, 2014). The issue included articles under several domains: theory, local practice, state practice and state policy, education, domain commentaries, and research, all intended to demonstrate life course theory in action and to give fresh perspectives and methods for tackling important problems in MCH by using a life course lens, per Pies.

The spread of this paradigm shift, through the means described above, as well as through leaders and community members alike, was an important historical moment in the MCH field and its recent evolution, worthy of remembrance and celebration. Advocates of life course theory didn’t know whether efforts would ultimately be successful since most work was still primarily theoretical or based in epidemiological research. As described by Fine and Kotelchuck, MCH practice had not perhaps changed to a similar degree, and some were skeptical or resistant to change.

### Changing MCH Practice

Translating theory into practice was a challenging endeavor, particularly where long standing programs were still perceived as important, in contrast to untested life course strategies that had long time horizons for impact. Advocates and leaders who endorsed life course perspective were challenged to show that the theory could lead to practices and programs that were more effective than those currently in place.

A key project encapsulating the movement of life course theory into practice was the Best Babies Zone (BBZ) initiative, originally conceived by Lu and further developed and led by Kotelchuck, Pies and other colleagues with funding from the W.K. Kellogg Foundation (Pies et al., 2016). The goal of the Initiative was to eliminate racial disparities in newborn mortality, through the creation of a transformed community—a Best Babies Zone—that addressed the perceived upstream roots of health outcomes. The key achievement of the Best Babies Zone approach was to operationalize life course theory by focusing on community-identified social determinants of health, and in doing so to mitigate exposure to risk factors and enhance protective factors for community well-being across generations. Starting in 2012 from a pilot in three communities, it grew to nine U.S. sites over the next seven years with a national office based at the University of California, Berkeley School of Public Health, where Pies served as principal investigator; the office offered continuous technical assistance, developed a national community of practice, and evaluated the initiative. The Best Babies Zone was grounded in four foundational strategies: place-based, multi-sectoral, community-driven, and aligned with broader social justice movements. Because health disparities arise from a network of systemic determinants, a multi-sector approach was used, including economic development, community systems and services, health systems, and education. Community-driven plans, with community priorities, were established in each Zone, recognizing the crucial input of lived experiences. The work was situated alongside and in support of broader national and grassroots political and policy struggles to advance racial, social, and reproductive justice, acknowledging that community transformation required collective effort. The Best Babies Zone’s adoption of an upstream strategy for community transformation acknowledged that achieving and measuring meaningful change would necessitate a long-term outlook (Reno et al., 2021). Pies recalled:

Measurement is so challenging. I don’t think there’s one formula. And the issues that are going to need to be addressed are going to be different in different environments. In one of the Best Babies Zone, they wanted to improve the neighborhood and blight, these were the issues that people wanted to work on…. If you’re thinking about birth outcomes, it seemed it had nothing to do in the moment with birth outcomes, but in the long term it did, and so those were the issues that were being raised by the local community. That’s how success was measured, by how we could respond to the community. It’s longitudinal… it’s the long term. So right now, we have to make those changes that will then impact and affect health later, and it’ll take a while to do that.

The Best Babies Zone community capacity and risk modifying approach was one of the first (since the early Children’s Bureau days) to try to reduce infant mortality with a focus on social and structural contexts. Its community-based organizing experiences, its local achievements, and integration of reproductive health with other community sectors (housing, nutrition, adult education, and employment) have all become part of current MCH initiatives. The Best Babies Zoon approach was a transformative life course intervention, that has now been replicated, adapted, and enhanced as a core component of broad community-based reproductive health initiatives as Kotelchuck noted.

### Other Perspectives

Neal Halfon’s work also provided a different perspective on making the theory actionable. In 2014 Halfon and colleagues (2014) updated the Life Course Health Development Framework to integrate newer findings from epigenetics, neurobiology, immunology, and epidemiology. They demonstrated how our understanding of health had changed over time from purely biological to biopsychosocial to longitudinal, as well as establishing that health is now understood to be a consequence of multiple determinants, including social, relational, environmental, policy and societal (Halfon et al., 2014). By regarding health as an active process that continues to develop throughout life, Halfon envisaged the future of the Life Course Health Development framework as a unifying approach that would enable multiple systems and sectors to work together towards the common goal of healthy development. This shifted the focus from surviving to thriving and highlighted the foundational importance of MCH for health across the whole of the life course, as Halfon described. The perspective places MCH firmly at the center of national health policy and suggests that it is seriously under-resourced, resulting in numerous missed opportunities for the active development of health capabilities in the early years. Halfon’s book on life course health development (Halfon et al., 2018) includes chapters that apply the life course framework to numerous contemporary MCH issues, that propose of seven principles of life course health development and their application to research and practice, and that describe an extensive research agenda. A Supplement to Pediatrics (Halfon & Hochstein, 2002) also included numerous papers on Advancing Life Course Intervention Research. In interviews Halfon described this:

The essential issue is that health develops over the life course, and this is a complex, adaptive system…life course health development emerges out of a complex developmental ecosystem… that led to the paper that I wrote on developing the 3.0 health system, because the 1.0 health system is built around the medical model. The 2.0 heath system is built around the biopsychosocial model and it’s about integration vertically, primary, secondary, tertiary care, horizontal. The 3.0 system is not about medical care or health care. It’s about health, and it’s about optimizing health over the lifespan, and it uses the life course health development model to design the system.

As noted, this period reflected growing experimentation with life course-informed interventions. The MCH Bureau, which was under the leadership of Michael Lu between 2012 and 2017, fostered such approaches, including for example targeted Infant Mortality Collaborative Improvement and Innovation Networks (CoIINs) devoted to social determinants of health. In addition, CityMatCH developed a series of urban infant mortality/reproductive health projects using the life course and Best Babies Zone model as part of its Advancing Birth Equity Strategies Together Cities initiative (Collie-Akers et al., 2021). Foundations, such as Robert Wood Johnson, WK Kellogg and others supported innovative community-based life course initiatives (as described by Parthasarathy). The pediatric community continued to undertake important life course interventions through the Center for the Developing Child at Harvard University (Shonkoff, 2003). HRSA/MCHB has continuously funded Life Course Network initiatives to further capacity, including a newly initiated Life Course Translational Research Network (LCT-RN) Program awarded to UCLA and led by Halfon. This research network aims to optimize life course health trajectories for children and families through integrating, rather than aggregating, the knowledge, skills, tools, and expertise that practitioners, youth and family members, community residents, researchers and other types of stakeholders can provide. The LCT-RN has an extensive network of members and active researchers in its “Nodes and Cores,” a Scholars program, and a growing record of NIH and Foundation funding for life course intervention research. The LCT-RN partners with communities, MCH and Title V services, and programs to co-design, test, implement, spread, and scale effective interventions in an effort to translate the benefits of the life course approach into practice.

## Discussion and Future Directions

As this oral history account has described, the life course perspective appealed to many in the MCH community from a personal, political, and experiential perspective, particularly those working in practice settings. Its growth and spread were fostered by advocates and policymakers, as well as committed MCH practitioners, and further substantiated by developments in research. After two decades, the formerly new ideas of life course theory have become a dominant paradigm in the field of MCH. As science has developed, so has the rationale for investment guided by a life course paradigm for equity. The current director of the MCHB, Michael Warren, recently described the commitments that the Bureau has made to integration of life course, noting:

The life course theory recognizes the vital role of social, economic, and environmental factors in influencing health outcomes, including health inequities. MCHB’s integration of a life course approach into multiple programs—including MIECHV, Healthy Start, research networks, and even the revised Title V framework—has furthered the Bureau’s longstanding work to address health inequities. (Warren & Kavanagh, 2023).

Life course is a key feature of national training MCH curricula and continues to be supported through academic workforce development programs (Deardorff et al., 2022; Haughton et al., 2013; Marshall et al., 2022). Teaching life course theory to MCH trainees is crucial, as it brings an opportunity to confront complexity and development. The theory also provides an excellent frame for introducing key social determinants of health concepts in the context of MCH; for example, trainees can engage in experiential learning about such determinants through the Life Course Game. Trainees may also see their own lives and family histories reflected in studying the life course approach, allowing them to make public health real in their own lived experiences.

In contemporary public health research and practice, social determinants of health and life course are considered basics tenets of MCH and health promotion in the United States (Braveman, 2014; Braveman & Barclay, 2009; Braveman et al., 2011; United States Department of Health and Human Services, 2023) as well as globally (World Health Organization, 2023). And yet, there is room for innovation and change, as Lu noted:

I think we need to continue to work on the science and do much as we did 20 years ago…questioning assumptions, asking the hard questions. I want to make sure we don’t get too comfortable with the life course perspective. I’m not sure we’re also listening enough to community voices. We have a responsibility to continue to think harder, to dig deeper, also to really identify the strategies, and not just the health care strategies, not just the public health strategies, but the social, economic, and political strategies based on the life course perspective.

The tragedy of Covid-19 and the crises caused by police and community violence, along with new restrictions on reproductive autonomy, have intensified the urgency of addressing racial disparities in MCH care and social determinants of health, indicating an evolving role for life course perspectives in future. The pursuit of reproductive justice necessitates innovative approaches to operationalizing the life course approach, alongside culturally appropriate standards for quality care, and a commitment to inclusion and diversity as essential components of transformational change in the MCH field.

Life course work today harkens back to founding concepts of the Children’s Bureau addressing social and economic drivers of health, and the impact of social class and inequity on mortality. To early MCH workers, maternity and childhood were a crucial period of growth and development, and it was recognized that the fight for their rights could not be won without social reforms (Yankauer, 1985). Cheri Pies reflected on the current moment this way:

When you think about George Floyd’s murder, or you think about other incidents that have happened across our country in the past four years or so, you see the implications not just of racial tension and justice, but you see the whole swath of social and economic injustice as well. Now there’s so much more attention to the issues of social determinants and racism, social and economic injustice. So, we’re in a different time. How life course gets implemented now, might be different. I think the work needs to have a passionate activist, an enthusiast, someone who is continually raising the issues and bringing the theoretical constructs to the fore to help people translate and relate that back to what we see on a day-to-day basis. I think you can’t have success without that.

The leaders who were interviewed for this oral history—and many others at the federal, state, and local level—brought life course thinking into the mainstream and created a movement which has changed teaching, research, practice, and policy in MCH. We encourage a new generation of MCH professionals to embrace the legacy and history of life course in order that the original Children’s Bureau mission will be carried forward.

## Data Availability

Not applicable.
